# Lead Emissions and Population Vulnerability in the Detroit Metropolitan Area, 2006–2013: Impact of Pollution, Housing Age and Neighborhood Racial Isolation and Poverty on Blood Lead in Children

**DOI:** 10.3390/ijerph18052747

**Published:** 2021-03-08

**Authors:** Heather A. Moody, Sue C. Grady

**Affiliations:** 1 Department of Environmental Science and Environmental Engineering, Siena Heights University, 1247 East Siena Heights Drive, Adrian, MI 49221, USA; 2 Department of Geography, Environment, and Spatial Sciences, Michigan State University, 673 Auditorium Road, Room 207, East Lansing, MI 48824, USA; gradys@msu.edu

**Keywords:** industrial lead, childhood blood lead levels, environmental justice, AERMOD, Detroit

## Abstract

This research investigates the relationships between airborne and depositional industrial lead emission concentrations modeled using Environmental Protection Agency’s (EPA’s) American Meteorological Society/Environmental Protection Agency Regulatory Model (AERMOD) and childhood blood lead levels (BLL) in the Detroit Metropolitan Area (DMA) 2006–2013. Linear and mediation interaction regression models estimated the effects of older housing and airborne and depositional lead emission concentrations on black and white childhood BLLs, controlling for neighborhood levels of racial isolation and poverty—important social structures in the DMA. The results showed a direct relationship between airborne and depositional lead emissions and higher childhood BLL, after controlling for median housing age. Lead emissions also exacerbated the effect of older housing on black and white children’s BLLs (indirect relationship), after controlling for social structures. Findings from this research indicate that black and white children exposed to lead-based paint/pipes in older housing are further impacted by industrial lead pollution that may lead to permanent neurological damage.

## 1. Introduction

This study is a continuation of research published in the IJERPH, 2017 [1] that found industrial lead emissions to be significantly higher in black (versus white) segregated neighborhoods, even after controlling for poverty. Lead emitting facilities and their emissions moved from black segregated communities of Pontiac in Oakland County to poorer and more black segregated communities of Wayne County, especially Dearborn, River Rouge, Ecorse, and Southwest Detroit. Today we ask, are the blood lead levels (BLLs) of children residing within these emissions elevated, controlling for housing age? Also, do children living in neighborhoods with older housing with greater lead pollution, experience a compounding risk after accounting for differences in neighborhood social structures?

This research adds to the historical environmental injustice literature of the Detroit Metropolitan Area (DMA), sharply divided by black and white race with almost 92% of whites living in the suburbs and 80% of all blacks living in Detroit itself [2]. Therefore, much research has been devoted to this dichotomy. Mohai and Bryant [3] showed that the proportion of black residents increased with geographic proximity to hazardous wastes and toxic releases in the DMA. Keeler et al. [4] found poor black communities in east and southwest Detroit were significantly burdened with more traffic particulate matter, while Wu and Batterman [5] revealed that non-white primary and secondary schools were located in impoverished neighborhoods of the city and exposed to significantly more auto/truck traffic emissions. Downey [6] also reported that black Detroit neighborhoods were disproportionately exposed to facility toxic release inventories. Subsequently, Lee and Mohai [7] determined that brownfield sites in the DMA were most likely sited adjacent to minority neighborhoods, especially African American communities, even after controlling for median income while Smith [8] showed that Superfund hazardous waste sites were strong predictors for geographic location in impoverished black neighborhoods in the DMA. Finally, Moody et al. [9,10] showed that within the DMA, decreasing socioeconomic position by neighborhood led to increasing BLLs for both black and white children but, black children residing in the same higher socioeconomic neighborhoods had higher BLLs than their white counterparts. The consensus of these studies is that race matters to exposure, even when controlling for socioeconomic indicators.

Lead is a well-established neurotoxin and carcinogen [11,12] and human exposure is most pronounced in old industrial urban environments. Sources include historically emitted leaded gasoline, nonferrous (brass and bronze) foundries, smelters, lead-acid-battery manufacturing, iron and steel production, power plants (refineries/coal burning), landfills, sewage sludge incinerators, waste incinerators, hazardous waste sites, and older homes that contain lead-based paint/pipes [12]. Lead emissions are common from industrial facilities lacking adequate air emission control equipment from coal burning power plants [13,14], coking operations [14,15], iron and steel manufacturing [16,17,18], oil refining [16,19,20], smelting [17,21,22], and waste incineration [16,17,23]. All these facility types, accompanied by emission deregulation, a lack of enforcement to existing regulatory limits, and limited air pollution control equipment, contribute airborne and deposition lead pollution to the DMA and especially the City of Detroit located in Wayne County [1]. Beginning the fall of 2012, Governor Rick Snyder’s Michigan Economic Development Corporation (MEDC) successfully lobbied the Michigan EPA for relaxed air permits for one of Detroit’s leading lead emitters, Severstal Steel (acquired by AK Steel in September 2014), who had been out of compliance with lead air emissions for years [24]. Further, Detroit Marathon Oil Refinery located in Southwest Detroit, also received a revised air permit allowing increased emissions, including lead, even though it too had unresolved air emissions violations [24,25]. Children living near these industrial facilities are most susceptible to lead poisoning because of their small size and developing physiology and may inhale lead from the air/dust and ingest lead from water and/or soil (dermal absorption is negligible) in places where lead accumulates [12].

Scientists have found no safe level of lead in children without doing permanent neurological and neurobehavioral harm [12,26,27,28,29,30,31,32,33,34]. Prior to January 2013, the CDC determined that 10 micrograms of lead per deciliter of blood (μg/dL) was a high level of concentration causing permanent neurological harm. In 2013, that level was changed to 5 μg/dL as the CDC continued to document the “no safe level” epidemiological evidence [12]. Therefore, there is a need to model this environmental contaminant associated with industrial activities alone, and the potential injustices in relation to location of residence.

Environmental injustice research methods suggest using a fine resolution of analysis for predicting pollutant exposure. Because race and low-income are inextricably linked, using dispersion models to incorporate weather, terrain, etc. while integrating this modeled data into GIS better estimates environmental injustice exposures geographically [35]. Modeled (AERMOD) industrial lead emissions (both airborne and depositional) were reported in IJERPH’s 2017 analysis [1]. This study is the first to our knowledge, to geospatially link these modeled emissions in GIS and relate the output to individual childhood BLLs.

The purposes of this research are to (1) characterize the BLLs of individual children at the address level in the DMA from 2006 through 2013, (2) overlay these BLLs on the already spatially modeled airborne lead and depositional concentrations (2006 through 2013) in the DMA, (3) relate these lead emissions to BLLs in children residing within, while controlling for housing age, and (4) assess the mediating interaction effects of housing age airborne and depositional lead emissions on childhood BLLs, controlling for social structures. It is hypothesized that neighborhoods receiving greater airborne and depositional industrial lead emissions will have greater childhood BLLs. Since it was previously shown that lead emitting facilities were more likely to be located in black segregated neighborhoods controlling for poverty [1], the detrimental effects of older housing age in those neighborhoods on childhood BLLs, are expected to be exacerbated by this additional airborne and depositional lead exposure. It is also hypothesized that living in older housing in neighborhoods with higher industrial lead emissions will have a similar effect on black and white children—after differences in neighborhood social structures are removed. Findings from this research will tease out the role uncontrolled/unregulated polluting industries have in poisoning our children by understanding other contributors in the environment such as leaded paint and pipes found within the home and neighborhood social structures. It can also provide a context for lead-hazard assessments at the individual and neighborhood level from which to remediate lead environmental exposure in the DMA.

## 2. Materials and Methods

### 2.1. Study Area

**Figure 1:**
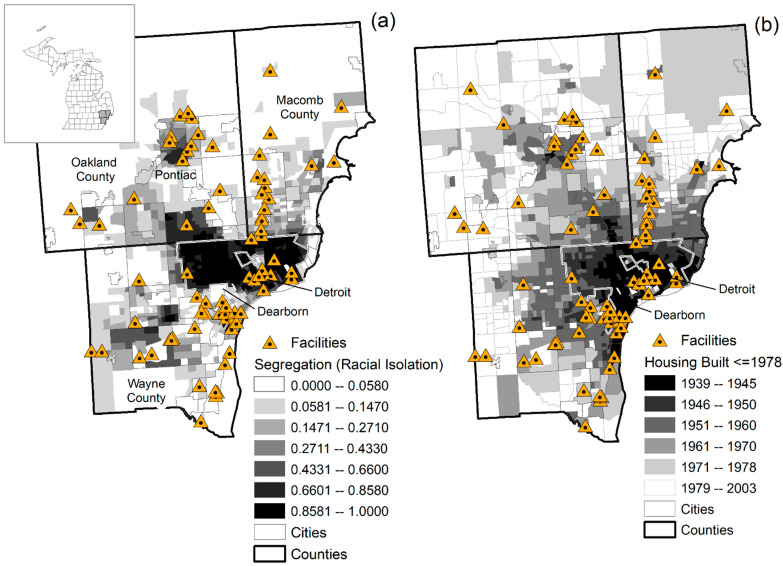
Locations of lead emitting facilities with city boundaries in the 3 County Detroit Metropolitan Area. (**a**) Black-white racial residential segregation (2010–2015) and (**b**) Housing Age Built during or before 1978 (1939–2003). This map was modified from the previously published map in reference [1]. Sources: American Community Survey (ACS) 2014 5-Year Estimate of Race, U.S. Bureau of the Census, 2020 [41]; ACS 2018 5-Year Estimates for Housing, U.S. Bureau of the Census, 2020 [42].

The study area remains the same as our previous IJERPH 2017 publication [1], conducted in the Detroit Metropolitan Area (DMA) of southeast Michigan, USA (Figure 1a,b). The DMA consists of three counties including Wayne, Macomb, and Oakland, equal to a population of 3,734,090 and an area of 2152 square kilometers (U.S. Census Bureau, ACS 2016) [36]. The City of Detroit is the center of the three-county urban cluster and is in Wayne County with a population of 713,862 in 2010 [36]—a 25.0% decrease from 2000 following the recession and housing foreclosure crisis [37]. The cities of Dearborn (population = 98,626) and Pontiac (population = 59,284), the latter located in Oakland County are the second and third largest cities in the DMA. The racial makeup of the DMA is 50% white, 38.5% black and 11.5% other [38]. Comparatively, the racial makeup of the City of Detroit has a high black population of 82.7 percent and white population of 10.6 percent including a large white-Arab population in the southwestern suburbs of Dearborn and Dearborn Heights [36]. In 2010, Detroit was also the poorest city in the country with 39.3% of residents living in poverty (Wayne County 24.0%, Macomb County 12.2%, and Oakland County 9.9%) [38]. The high percentage of black population and low income of residents in Detroit reflects a concentration of poverty—i.e., racially segregated neighborhoods with severely disadvantaged social environments and reduced life chances [39,40] increasing vulnerability to poor health. Figure 1b also shows that older housing is concentrated in highly segregated areas of the City of Detroit (Wayne County) and surrounding cities to the southwest of Wayne County and north into Pontiac (Oakland County) and Macomb County showing the extent of older housing stock in the DMA. Newer housing is situated in less segregated areas of suburban DMA and further beyond in mostly white rural areas where housing is again older but not to the extent as in Detroit and adjoining cities.

### 2.2. Childhood BLLs

Childhood blood lead levels (BLLs) were obtained from the Michigan Department of Health and Human Services (MDHHS) for the Detroit Metropolitan Area for the years of study (2006–2013). De-identified values, provided as integers in micrograms per deciliter of blood (ug/dL), were accompanied by addresses. Due to the CDC’s policy of recording non-detect lead levels as 1 μg/dL, methodological guidance has been issued by the Department of Health and Human Services, Centers for Disease Control and Prevention to transform the values to geometric means to accommodate for these values [43]. The MDHHS values also included the child’s sex, age in days (converted to years) from 0 to 16 years, race/ethnicity (self-reported by parent), type of health insurance, and blood collection method type. There were children who were BLL tested using the capillary and venous methods. If an elevated BLL presented in the first capillary or venous test (≥10 μg/dL during the dates of this study), the child was administered a second venous test and only this new venous BLL was entered. All duplicate test results of the same child over the period of years studied were removed, keeping the highest BLL value. The DMA screening rate (proportion of children BLL tested divided by the census 2006–2010 5-year estimate of the childhood population as a whole under 5 to 14 years) was 0.53 [44]. A high proportion (72.0%) of children with BLL tests in the MDHHS dataset were Medicaid recipients.

### 2.3. Lead Emissions and Modeling

See Appendix A for AERMOD lead emissions modeling.

### 2.4. Modeled Lead Emissions and Recipient Individuals’ BLLs

This study utilizes the modeled total deposition (wet plus dry) and airborne concentrations from the 2017 study [1]. The 500 by 500-m lead concentration grids were mapped in ArcGIS and joined with the geometric mean BLLs of individual children at their address using version 10.6 [45]. The relationship among these variables was explored using Pearson’s correlation and bivariate linear regression at the individual level. It was hypothesized that both a positive correlation and slope relationship between wet and dry deposition and individual BLLs would result. It was also hypothesized that both a positive correlation and slope relationship between airborne concentrations and individual BLLs would result.

Because the greatest predictor of lead poisoning in children is an older home environment (lead house paint both interior and exterior, pipes, fixtures, etc.) [46], controlling for housing age is important. A mixed effects logistic regression model was used to assess associations between lead emissions, childhood BLLs, and industrial lead pollution. Both wet and dry depositional lead from industry will lay down and combine with lead laden soils and dust in and outside the home confounding the exposure. Lead based paint has been banned from use in residential homes starting in 1978 and from plumbing systems in 1986 [47]. Given this date and to isolate the exposure to industrial lead emissions alone, a housing age interactive term was added. All housing ages (median year structure built from The U.S. Bureau of the Census, American Community Survey) by census tract [44] were classified and geocoded to the individual children’s address of residence. If the median year the homes were built were after 1978, the census tract was classified as zero and those built on or before were classified as 1. Total deposition and then airborne deposition were multiplied by this code and regressed against geometric mean BLLs controlling for gender and race/ethnicity. It was hypothesized that as the wet and dry depositional values with the added housing age interaction term increased, the BLLs of the children within those census tracts would increase. This was also hypothesized for airborne lead concentrations. We used SPSS version 25 (IBM, Armonk, NY, USA) for the analyses and maps of total deposition, airborne deposition, and childhood BLLs were generated in ArcGIS version 10.6. [45]. To protect individual children’s BLL by address, they were aggregated and averaged at the census tract level on maps below.

### 2.5. Causal Mediator Interaction Models

Racial residential segregation was modeled using a multilevel modeling approach in MlwiN v. 3.02 (University of Bristol, Bristol, UK) [48] to estimate the proportion of blacks within census tracts, controlling for area-level poverty. A two-level binomial distribution with a logit link function was used to model the mean proportion of blacks at level-1 (fixed effects) and the level-2 variation in proportion blacks at the census tract level (random effects), controlling for census tract level poverty. The predicted proportion black was multiplied by the total population to obtain the predicted number of blacks and number of other racial and ethnic groups. The black and other-group calculations were then input into STATA v. 13.1 (StataCorp, Lakeway, TX, USA) [49] to compute a black isolation index at the census tract level using the ‘SEG’ module [50,51]. Racial isolation refers to the likelihood that blacks would come into contact with other racial and ethnic groups in their census tract of residence. The race and poverty data and median housing age at the census tract level were obtained from the U.S. Census Bureau’s American Community Survey (ACS) 2010–2014 and as described above [41].

**Figure 2:**
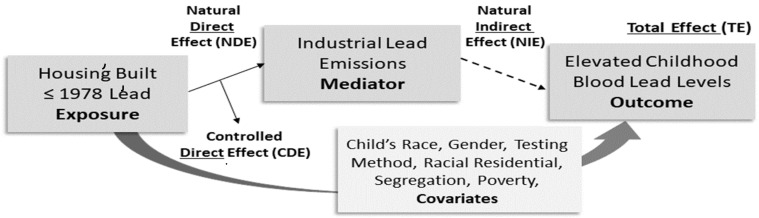
Causal mediation interaction effects of airborne lead and industrial emissions for children living in housing built ≤ 1978 on childhood blood lead levels.

Causal mediator interaction models were constructed to evaluate the effect of children living in housing built ≤ 1978 (exposure) on childhood BLLs (outcome) and the effects of industrial lead emissions (mediator) in that relationship. See Figure 2. The model’s total effects (TE) are the natural direct and indirect effects (NDE + NIE). The model’s controlled direct effects (CDE) are the effect of housing built ≤ 1978 on childhood BLLs. The Natural Direct Effect (NDE) = CDE + the reference interactive between housing built ≤ 1978 + industrial lead emissions. The Natural Indirect Effect (NIE) = the mediating effect of industrial lead emissions on childhood BLL. The percent mediation (PM) = NIE/TE × 100%. The percentage of the effect explained by the interaction (PAI) = NDE/TE × 100%. The percent eliminated (PE) = CDE/TE × 100% [52].

## 3. Results

### 3.1. Study Area

Figure 1a,b are reference maps showing the AERMOD domain and locations of lead emissions facilities. Of the 78 stationary facilities emitting lead, 41 (52%) were in Wayne County, 19 (24%) were in Oakland County and 17 (22%) were in Macomb County. There were 12 facilities in the city of Detroit and 15 facilities on the western and southwestern periphery of Detroit representing 66% of facilities in Wayne County. In Oakland County the facilities were concentrated around the cities of Pontiac, Novi, Wixom, Auburn Hills, and Rochester Hills. In Macomb County the lead emitting facilities followed the north-south State Highway M-53. These facilities in general extended across the study domain. Of these 78 facilities, 48 (61.5%) had more than one emissions stack or fugitive source (an industrial process not captured or vented through a stack). Unlike the federal EPA Toxic Release Inventory data requirements for emissions reporting, the MAERS state data must enter a quality assurance/quality control program and is based on the lead emission activity and devices of each facility’s point sources (usually stacks) [53].

### 3.2. Childhood BLLs

**Figure 3:**
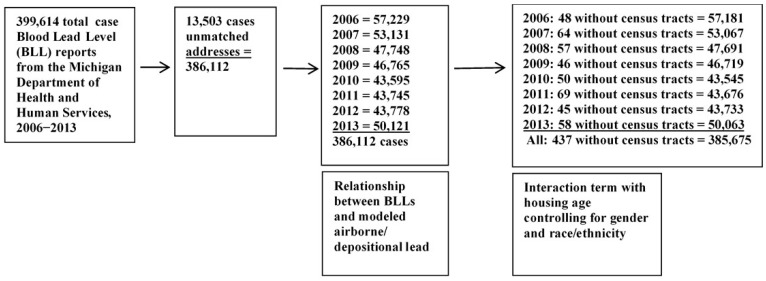
Flow diagram of data retrieved from the overall dataset. Case report numbers were queried to provide only one test for each child during this time period.

The BLL addresses were geocoded to roads (within census tracts of the DMA) and achieved a 99.3% match rate. There were 154,902 “matches” (all with at least a score of 90, most a score of 100). The remaining 1151 were “tied” with scores of at least 90. Figure 3 below displays the number of original BLL cases geocoded by address and then geocoded to census tracks.

A summary of the BLLs for the children of the DMA are displayed on Table 1 by year. It is notable that over the 8-years, geometric mean BLLs have declined and especially the percent of cases with ≥5 µg/dL. As supported by the literature on exposure [12], male, non-Hispanic Black, and Medicaid children had a higher percentage of BLLs than their counterparts. The 2.1—6-year age grouping had the highest percentage likely due to floor activity and hand-to-mouth behavior, also established in the literature [12]. The intravenous blood collection method is preferred due to greater accuracy.

### 3.3. Lead Emissions and Modeling

Pounds of lead emitted from industrial stacks increased for the first three years and then began to drop rapidly in Macomb and Oakland counties, but not for Wayne County and the City of Detroit (see Table 1). One thousand plus pounds per year occurred roughly throughout the entire study period for Wayne County/Detroit and by 2013, began to rise near the 2006 levels. This can be largely attributed to the relaxed air permitting allowed of AK Steel in Dearborn and the Marathon Oil Refinery located in southwest Detroit under Governor Snyder’s administration [1]. The mean modeling results from these emissions can also be found in Table 1. As reported in the IJERPH 2017 study [1], those areas with the highest accumulation of lead (airborne and depositional) were in the mostly black segregated communities, especially those of Detroit and southwest Detroit.

### 3.4. Relationship between Industry Lead Emissions and BLLs

**Figure 4:**
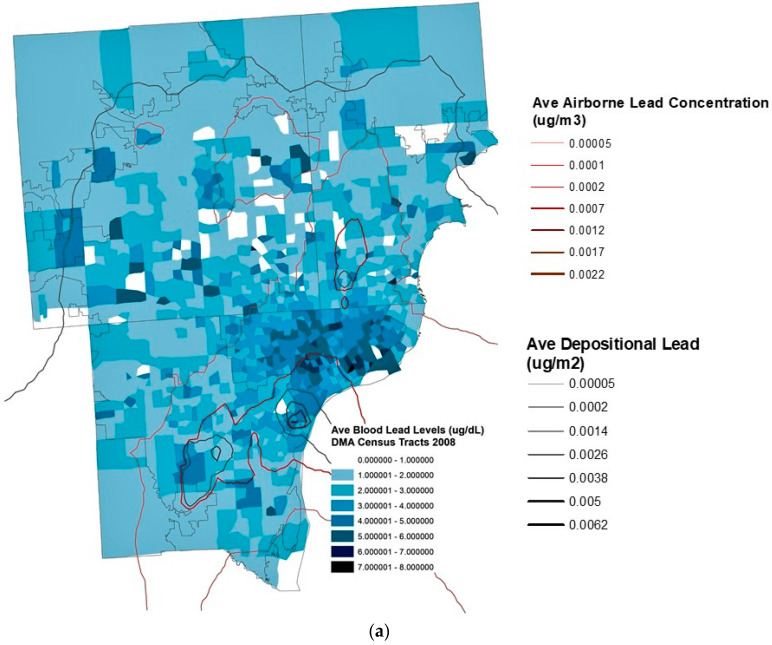

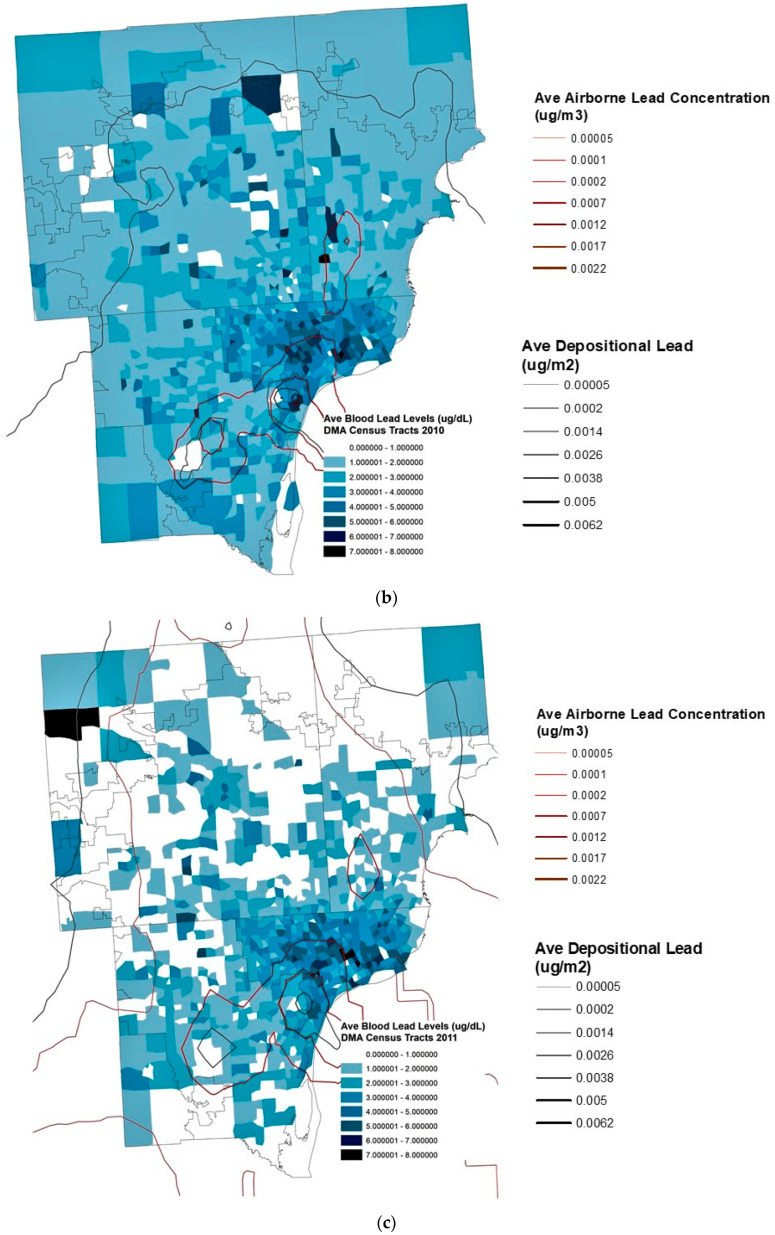
(**a**) Detroit Metropolitan Area Average Blood Lead Levels (BLLs) by Census Tract. These Airborne/Depositional Concentrations Strongly Correlated/Regressed with Childhood BLLs 2008. (**b**) Detroit Metropolitan Area Average Blood Lead Levels (BLLs) by Census Tract. These Airborne/Depositional Concentrations Strongly Correlated/Regressed with Childhood BLLs 2010. (**c**) Detroit Metropolitan Area Average Blood Lead Levels (BLLs) by Census Tract. These Airborne/Depositional Concentrations Strongly Correlated/Regressed with Childhood BLLs 2011.

Adding to the industrial lead emission modeling from the IJERP 2017 study [1], total industrial lead deposition emissions, both wet and dry, are positively and significantly correlated with children’s individual BLLs. The depositional material falls as dust and accumulates in and around homes. Airborne lead emissions also positively and significantly correlate with children’s individual BLLs but at twice the coefficients. These emissions stay airborne in a gaseous state. Noteworthy are the years 2008, 2010, and 2011 with the largest correlation slope coefficients of deposition/airborne to BLLs; 0.087/0.092 (*p* = 0.000), 0.139/0.115 (*p* = 0.000), and 0.059/0.067 (*p* = 0.000) respectively. As depositional and airborne concentrations of lead increased, childhood BLLs increased significantly so. See Table 2 and Figure 4.

Bivariate regression of lead deposition/airborne emissions with individual BLLs, yielded a significant positive relationship with large coefficients, especially for the years 2008, 2010, and 2011 yielding slope coefficients of 59.698/361.895 (*p* = 0.000), 132.029/564.149 (*p* = 0.000), and 54.003/306.202 (*p* = 0.000) respectively. Airborne lead emissions were very strongly positively correlated to individual childhood BLLs producing ten times greater coefficients than deposition. For every unit increase in airborne lead in 2010, a 564.149 unit increase in BLLs occurred. See Table 3 and Figure 4.

When attempting to control for housing age using continuous raw numbers, confounding and unacceptable tolerance levels occurred especially when related to total lead deposition that combines with dusts of older interior/exterior housing paint. In order to disentangle all sorts of lead stressors in the child’s environment, the housing age interaction term regressed with BLLs (controlling for gender and race/ethnicity) proved significant in the positive direction with even larger coefficients, especially in regard to airborne lead emissions. The greatest effect occurred in 2010 with a depositional regression slope coefficient of 144.503 (*p* = 0.000) and an airborne regression slope coefficient of 577.506 (*p* = 0.000). All coefficients were larger with the interaction term; older housing is thus shown to have an effect, in addition to lead emissions, on childhood BLLs. See Table 3.

Figure 4 shows BLLs of the children tested, geocoded by address and spatially joined to census tracts and within proximity of the modeled depositional and airborne lead values. Airborne and depositional breakpoints were established from the entire years data, starting at the lowest concentration and up to the highest, then evenly divided within. Visually, it is apparent that higher BLLs cluster around areas of heavier modeled lead emissions and their sources. The 2008, 2010, and 2011 series of maps were chosen because those years yielded the strongest airborne and depositional correlation/unadjusted regression coefficients associated with childhood BLLs (see Table 2 and Table 3). A marked decline in childhood BLLs is apparent overall but accompanied by persistent concentrations in Detroit and southwest Detroit.

### 3.5. Causal Mediator Interaction Models

Examining the causal mediator and interaction models for the three highlighted years above, the study found that children living in older housing were significantly more likely than children living in newer housing to have higher BLLs, controlling for differences in neighborhood social structure. Children exposed to increasing depositional lead experienced significantly higher BLLs in 2008 and 2010 (see Table 4). Depositional lead explained 54.4% (range, 50.0–58.4%) of the total model effects in 2008, and 44.3% (range, 40.7–48.0%) in 2010. Depositional lead significantly exacerbated the effects of older housing on children’s BLLs with its greatest impacts in 2008 followed by 2010 and 2011. Children exposed to increasing airborne lead concentrations also had significantly higher BLLs in 2008 and 2010 as well as 2011. Airborne concentrations explained 56.5% (range, 52.2–60.8%) of the total model effects in 2008, 43.9% (range, 40.4–47.4%) in 2010 and 16.3% (range, 11.9–20.7%) in 2011. Airborne lead concentrations significantly exacerbated the effects of older housing on children’s BLLs with its greatest impacts also in 2008, 2010 and 2011. Airborne lead concentrations persisted as a significant risk factor for childhood BLLs across all three time periods. See Table 4.

Importantly, black and white children exposed to increasing depositional lead or airborne lead concentrations experienced significantly higher BLLs in 2008, 2010, and 2011 (see Table 5). Depositional lead explained black-61.0% (range, 55.7–66.3%) and white-56.3% (range, 51.7–60.9%) of the total model effects in 2008, in 2010 black-49.3% (range, 44.9–53.8%) and white-50.6% (range, 45.9–55.2%) and in 2011 black-11.9% (range, 5.6–18.3%) and white-11.9% (range, 5.6–18.2%). Depositional lead exacerbated the effects of older housing on black and white children’s BLLs across all time periods, with slightly greater effects on white compared to black children in 2008 and black compared to white children in 2010. Airborne lead concentrations explained black-83.4% (range, 75.5–91.2%) and white-77.8% (range, 70.9–84.8%) of the total model effects in 2008, in 2010 black-55.2% (range, 50.5–59.9%) and white-53.7% (range, 49.2–58.1%) and in 2011 black-31.3% (range, 25.9–36.8%) and white-30.2% (range, 25.2–35.2%). Airborne lead concentrations exacerbated the effect of older housing on black and white children’s BLLs with its greatest effects in 2008 followed by 2010 and 2011. See Table 5.

Referring to airborne lead factors in 2008, 2010, and 2011, the total effect models showed that black and white children had significantly high BLLs that were in part explained by older housing and airborne lead emissions while controlling for social structures. Older housing was a significant risk factor for increasing BLLs for black and white children. Likewise, airborne lead was also a significant mediator in the housing age and childhood BLL relationship, similar in 2008 and 2010 but lessening in 2011. Airborne lead explained a substantially higher percentage of BLLs for black children in 2008 and slightly higher percentage of BLLs for black children in 2010 and 2011 compared to white children. The percentage of the interaction between older housing and airborne lead on BLLs was higher for white children in 2008 and 2010 compared to black children but the results were relatively similar in 2011.

## 4. Discussion

Over the study period, 2007–2008 had the highest overall lead emissions followed by the year 2010. In 2008, the largest lead emitters were located in southwest Detroit and Dearborn (Marathon Oil and AK Steel, respectively) and were granted permission to operate under 2006 permit levels, increasing the quantities of lead emitted in these cities and communities [1]. We already know from the 2017 IJERPH Moody and Grady [1] study that lead emissions during this time were significantly higher in racially isolated black communities of Detroit overall, and southwest Detroit in particular. Dearborn to the west of southwest Detroit is 90.2% white with a large (40%) Arabic population [54]. This study showed that depositional and airborne lead contributed directly and significantly to children’s BLLs, even after controlling for housing age and social structures, with black children at a continued disadvantage. Further analysis revealed that children residing in older housing in neighborhoods with high levels of airborne and depositional lead had a compounding risk of higher BLLs, and this effect was observed for black and white children, after controlling for differences in their neighborhood social structures. These findings demonstrate that racial disparities in childhood BLLs are largely explained by exposures to older housing age and high airborne or depositional lead pollution in the DMA (appearing to be driven by lead emitting industries in Detroit and Dearborn). This study adds to the environmental injustice literature by showing that black and white race matters, and when ethnic origin is further considered, as in this case, a large Arab population of children in Dearborn.

Maantay [55] defines environmental justice as, at a minimum “rights for all people to live in a healthy environment, one free from hazardous conditions and noxious substances, and adherence to the goal that no specific population should bear a disproportionate burden of the waste products of modern life and industry.” (p. 9) Maantay states that some definitions go further to include unequal enforcement and implementation of environmental laws and regulations and others emphasize uneven geographical locations of pollution, which this study does. She warns that environmental justice should not mean that we are all exposed evenly either, but rather a reduction/elimination of pollution all together. This study by no means accepts that anyone should live with lead pollution even in the guise of Governor Snyder’s historical de-regulation and un-resolved lead violations of AK Steel (formally Severstal Steel) and Marathon Oil in ethnic communities. Nor should it go unmentioned that the unmet EPA cleanup standards of southwest Detroit’s NL Industries Master Metal smelter pollution need attention [1]. As shown, regulating lead emissions matter. Enforcement of these regulations and adequate air emission control equipment as required of other wealthy countries would largely prevent exposure to all groups. Other major contributors such as DTE’s coal burning facilities within the DMA are largely unnecessary given the ease and cost of solar/wind electric energy technologies.

Limitations exist when using specific industrial emissions reports and AERMOD. The modeled lead emissions data came from industries required to report to MAERS but only if they are a listed lead industrial activity as established by the State. The MAERS emissions data do not list all sources of lead air pollution such as small contributors not obligated to report [56]. Also, the modeled emissions are only estimated for each facility’s lead emitting activity regulated. AERMOD incorporates these annual seasonal emission rates and applies them over a 12-month period to quantify monthly and annual concentrations. As yet, the EPA has not published estimates of error or confidence intervals surrounding AERMOD parameters or output values. However, this model is the best analytical source available and has been tested extensively prior to adoption as a regulatory tool. Findings from this research show the role of uncontrolled/unregulated polluting industries in poisoning children. Another limitation is the very large number of children (*n* = 386,112) tested for BLLs and the large number of AERMOD airborne/depositional grid cells (average *n* = 49,500). Nuances in the relationships/coefficients (*p*-values) may be lost due to such large numbers.

Another limitation is the large number of individual childhood BLL reports and lead pollution grid cells. Small geographic study would yield more sensitive coefficients/*p*-values and allow for using mean housing age as a continuous variable, perhaps modelling lead emissions as a contextual rather than individual-level exposure. This would also enable finer geographic map representation and eliminate the need to select only those years of greatest significance.

Regardless, noxious lead pollution is directly poisoning, irreparably, children of those areas in the DMA with older housing and lead emissions. Many of the most impacted neighborhoods have fought for environmental justice and against structural racism for decades. As in many urban centers across the United States, the structural racism of segregation is by design and specifically, racial residential segregation exposes people of color (here, black race and ethnic origin) to disparate and negative environmental hazards including industrial pollutants, waste disposal/incineration contaminants, placement of interstate highways with accompanying truck emissions, etc. [57]. Lead emitting industries prefer locating in neighborhoods concentrated by people of color, people with limited political and economic power. This pattern is “a manifestation of deeply rooted institutional racism” [58] (p. 321).

Predicted potential IQ loss of these children is notable but does not make their lives disposable. In a 2017 longitudinal *JAMA* study, Reuben and others found that of 565 participants in a Dunedin Multidisciplinary Health and Development Study of New Zealand, 11-year-olds with increasing BLLs (by increments of 5 µg/dL), experienced a significant 1.6-point reduction in IQ by the age of 38. A BLL of 10 µg/dL yielded a 2.7-point IQ reduction by the age of 38 [59]. This study of the DMA found that in 2006 alone, there were 104 children tested with BLLs ranging from 5–9 µg/dL, reducing their adult (38 years old) IQ by 1.6 points. Another 11 children had BLLs from 10–56 µg/dL potentially reducing their adult IQ by 2.7 at least. This predicted IQ loss model likely underestimates the damage as additional research is revealing even greater IQ impacts of children living with socioeconomic disadvantage.

Additional research should emphasize enforcement of lead polluting industries, while also addressing older housing remediation in the study of racial and ethnic disparities in childhood BLLs. Future study will include the findings from this manuscript in relation to its contribution to maternal exposures and adverse birth outcomes. Further investigation into the racial and ethnic composition of residents in black and white residentially segregated areas could also help to disentangle the detrimental effects of racial isolation as experienced by black residents and children, and the ethnic density effect that exhibits protective health effects by living among residents of similar culture and backgrounds [60,61]. However, regardless of these neighborhood social structures, this study found that childhood exposure to older housing in areas targeted for environmental injustice, here due to high airborne and depositional lead pollution from emitting industries on BLLs, is an area in need of continued research.

## 5. Conclusions

This study found that from 2006 to 2013, children living within industrial lead emissions, as modeled, were directly impacted with higher BLLs. Interventions are needed to prevent neurological, permanent damage. A continuing trend of weakening permit conditions allowing for increasing lead emissions in these neighborhoods, combined with a lack of enforcement power, continue to subject black and white (ethnic) children to greater exposure, demonstrating environmental injustices in the DMA. These findings should be used to stop lead emissions, or at least require adequate air emission control equipment along with intervention and to inform current and future regulators as to the unjust and largely preventable nature of lead exposure to children in the Detroit Metropolitan Area.

## Figures and Tables

**Table 1:** Characteristics of Detroit Metropolitan Area (DMA) Children’s Geometric Blood Lead Level (BLL) ^†^ Reports and Lead Emissions/Modeling Across Years 2006–2013.

Variables								
	2006	2007	2008	2009	2010	2011	2012	2013
Sex								
Male = 0	29,722 (51.90)	27,701 (52.10)	24,726 (51.80)	24,101 (51.50)	22,426 (51.40)	22,544 (51.50)	22,739 (51.90)	25,759 (51.40)
Female = 1	27,507 (48.10)	25,430 (47.90)	23,022 (48.20)	22,664 (48.50)	21,169 (48.60)	21,201 (48.50)	21,039 (48.10)	24,362 (48.60)
Total/Mean/Std Dev	57,229/0.48/0.50	53,131/0.48/0.50	47,748/0.48/0.50	46,765/0.48/0.50	43,595/0.49/0.50	43,745/0.48/0.60	43,778/0.48/0.50	50,121/0.49/0.50
Age in Years								
0–2	14,419 (25.20)	16,066 (30.23)	16,866 (35.32)	18,800 (40.20)	19,025 (43.64)	21,500 (49.15)	22,662 (51.77)	27,615 (55.10)
2.1–6	33,600 (58.71)	28,375 (53.41)	23,803 (49.85)	22,000 (47.04)	19,734 (45.27)	18,365 (41.98)	18,045 (41.22)	19,888 (39.68)
>6 to 16	9210 (16.09)	8690 (16.36)	7079 (14.83)	5965 (12.76)	4836 (11.09)	3880 (8.87)	3071 (7.01)	2618 (5.22)
Total/Mean/Std Dev	57,229/4.04/2.85	53,131/3.87/2.91	47,748/3.65/2.97	46,765/3.37/2.88	43,595/3.19/2.81	43,745/2.91/2.69	43,778/2.71/2.53	50,121/2.48/2.23
Race/Ethnicity								
Non-Hispanic Black = 1	26,090 (45.60)	21,452 (40.40)	16,769 (35.10)	15,939 (34.10)	15,455 (35.50)	15,229 (34.80)	15,694 (35.80)	18,283 (36.50)
Non-Hispanic White = 2	17,358 (30.30)	18,181 (6.60)	18,921 (39.60)	19,261 (41.20)	18,131 (41.60)	18,179 (41.60)	17,684 (40.40)	20,299 (40.50)
Hispanic = 3	4030 (7.00)	3500 (6.60)	2875 (6.00)	2530 (5.40)	2353 (5.40)	2386 (5.50)	2487 (5.70)	2741 (5.50)
Native Hawaiian or Pacific Islander = 4	64 (0.10)	57 (0.10)	60 (0.20)	67 (0.10)	97 (0.20)	144 (0.30)	150 (0.30)	285 (0.50)
Asian = 5	605 (1.10)	693 (1.30)	765 (1.60)	824 (1.80)	813 (1.80)	959 (2.20)	904 (2.10)	1162 (2.30)
Arab = 6	153 (0.30)	No Longer Reported	No Longer Reported	No Longer Reported	No Longer Reported	No Longer Reported	No Longer Reported	No Longer Reported
American Indian or Native Alaskan = 7	28 (0.00)	143 (0.30)	159 (0.40)	184 (0.40)	186 (0.40)	138 (0.30)	136 (0.30)	180 (0.40)
Mixed Race = 8	28 (0.00)	19 (0.00)	16 (0.00)	11 (0.00)	35 (0.10)	28 (0.10)	43 (0.10)	54 (0.10)
No Report = 9	8901 (15.60)	9086 (17.10)	8183 (17.10)	7949 (17.00)	6525 (15.00)	6682 (15.20)	6680 (15.3)	7117 (14.20)
Total	57,229	53,131	47,748	46,765	43,595	43,745	43,778	50,121
BLL µg/dL Cases	57,229 (100.00)	53,131 (100.00)	47,748 (100.00)	46,765 (100)	43,595 (100)	43,745 (100)	43,778 (100)	50,121 (100)
† Mean/Stan Dev	3.05/2.98	2.78/2.69	2.50/2.75	2.43/2.59	2.28/2.54	2.15/2.18	2.00/2.05	2.00/2.09
% ≥5 µg/dL	17.38	14.22	10.67	9.21	7.42	5.65	5.21	4.37
Blood Collection Method								
Capillary = 0	9886 (17.30)	8389 (15.80)	9225 (19.30)	10,727 (22.90)	10,642 (24.40)	12,225 (27.90)	13,474 (30.80)	18,678 (37.30)
Intravenous = 1	47,343 (82.70)	44,742 (84.20)	38,523 (80.70)	36,038 (77.10)	32,953 (75.60)	31,520 (72.10)	30,304 (69.20)	31,443 (62.70)
Total/Mean/Std Dev	57,229/0.83/0.38	53,131/0.84/0.37	47,748/0.81/0.40	46,765/0.77/0.42	43,595/0.75/0.43	43,745/0.72/0.45	43,778/0.69/0.46	50,121/0.63/0.48
1.0 µg/dL Reports	16,365 (28.60)	18,199 (34.25)	20,029 (41.95)	17,776 (38.01)	18,555 (42.56)	18,130 (41.44)	23,885 (54.56)	26.507 (52.89)
Insurance Coverage Type								
Non-Medicaid = 0	12,867 (22.50)	14,341 (27.00)	14,585 (30.50)	14,120 (30.20)	10,642 (24.40)	12,951 (29.60)	12,119 (27.70)	13,559 (27.10)
Medicaid = 1	44,362 (77.50)	38,790 (73.00)	33,163 (69.50)	32,645 (69.80)	32,953 (75.60)	30,794 (70.40)	31,659 (72.30)	36,562 (72.90)
Total/Mean/Std Dev	57,229/0.78/0.42	53,131/0.73/0.44	47,748/0.69/0.46	46,765/0.70/0.46	43,595/0.71/0.45	43,745/0.70/0.46	43,778/0.72/0.45	50,121/0.74/0.44
Total Pounds Lead Emitted								
Wayne County (Detroit)	1483 (68.03)	1809 (69.95)	1559 (46.89)	1051 (59.28)	981 (88.62)	930 (93.28)	1242 (98.26)	1431 (99.17)
Macomb County	55 (2.52)	87 (3.36)	38 (1.14)	30 (1.69)	9 (0.81)	15 (1.50)	6 (0.47)	1 (0.07)
Oakland County	642 (29.45)	690 (26.68)	1728 (51.97)	692 (39.03)	117 (10.57)	52 (5.22)	16 (1.27)	11 (0.76)
Total:	2180	2586	3325	1773	1107	997	1264	1443
Mean Modeled Total Lead								
Deposition, DMA (µg/m^2^)	0.00225	0.001164	0.00134	0.000867	0.001237	0.001197	0.001966	0.002113
Mean Modeled Airborne								
Emissions, DMA (µg/m^3^)	0.000994	0.000244	0.000324	0.000145	0.000296	0.000206	0.000378	0.000507

† Geometric Mean BLL, *n* (%); Underlines = summation.

**Table 2:** Covariate Relation between Lead Emissions and Geometric Mean Blood Lead Levels (BLLs) in the Detroit Metropolitan Area, 2006–2013.

	2006	2007	2008	2009	2010	2011	2012	2013
Modeled Total Lead Deposition (Wet plus Dry) (µg/m^2^)	0.022 *n* = 57,229	0.047 *n* = 53,131	0.087 *n* = 47,748	0.047 *n* = 46,765	0.139 *n* = 43,595	0.059 *n* = 43,745	0.037 *n* = 43,778	0.046 *n* = 50,121
Modeled Airborne Lead Emissions (µg/m^3^)	0.048 *n* = 57,229	0.059 *n* = 53,131	0.092 *n* = 47,748	0.080 *n* = 46,765	0.115 *n* = 43,595	0.067 *n* = 43,745	0.066 *n* = 43,778	0.062 *n* = 50,121

Data represent the modeled coefficients of the relation between lead deposition (µg/m^2^) and airborne lead (µg/m^3^) and childhood BLLs (µg/dL). *n* = 386,112 across all years; all coefficients were highly significant (*p* = 0.000) Source: Michigan Department of Health and Human Services 2006–2013 and MAERS 2006–2013.

**Table 3:** Bivariate/Multivariate Relation Between Lead Emissions and Geometric Mean Blood Lead Levels (BLLs) in the Detroit Metropolitan Area, 2006–2013.

	2006	2007	2008	2009	2010	2011	2012	2013
Modeled Total Lead Deposition (Wet plus Dry) (µg/m^2^)	24.82 *n* = 57,229	41.853 *n* = 53,131	59.698 *n* = 47,748	23.472 *n* = 46,765	132.029 *n* = 43,595	54.003 *n* = 43,745	24.507 *n* = 43,778	43.982 *n* = 50,121
Modeled Airborne Lead Emissions (µg/m^3^)	258.727 *n* = 57,229	236.26 *n* = 53,131	361.895 *n* = 47,748	354.539 *n* = 46,765	564.149 *n* = 43,595	306.202 *n* = 43,745	291.275 *n* = 43,778	293.662 *n* = 50,121
Interaction Term								
Total Deposition	60.764	74.576	77.015	77.504	144.503	80.85	44.937	69.053
Lead * Housing	−0.024	−0.026	−0.013	−0.015	−0.017	−0.014	−0.014	−0.010
Gender	−0.027	−0.024	−0.016	−0.007	−0.008	−0.006	−0.007	−0.007
Race/Ethnicity	*n* = 57,181	*n* = 53,067	*n* = 47,691	*n* = 46,719	*n* = 43,545	*n* = 43,676	*n* = 43,733	*n* = 50,063
Interaction Term								
Airborne Lead *	392.084	328.556	423.139	435.933	577.506	314.628	383.016	384.383
Housing	−0.024	−0.026	−0.013	−0.015	−0.017	−0.014	−0.014	−0.010
Gender	−0.027	−0.024	−0.016	−0.007	−0.008	−0.005	−0.007	−0.007
Race/Ethnicity	*n* = 57,181	*n* = 53,067	*n* = 47,691	*n* = 46,719	*n* = 43,545	*n* = 43,676	*n* = 43,733	*n* = 50,063

Data in rows 1 and 2 represent the modeled coefficients of the relation between lead deposition (µg/m^2^) and airborne lead (µg/m^3^) and a change of 1 µg/dL in childhood BLLs. Data in rows 3 and 4 represent the modeled coefficients of the relation between lead deposition (µg/m^2^) * housing age and airborne lead (µg/m^3^) * housing age and a change of 1 µg/dL in childhood BLLs controlling for Gender and Race/Ethnicity. All coefficients were highly significant (*p* ≤ 0.001) Source: MDHHS 2006–2013, MAERS 2006–2013, and American Community Survey 2006–2010 5-year Estimates.

**Table 4:** Causal Mediation and Interaction Effects of Older Housing (Built ≤ 1978) and Average Depositional Lead or Average Airborne Lead Concentration on Childhood Blood Lead Levels, Controlling for Racial Residential Segregation, Detroit Metropolitan Area, 2008, 2010, 2011.

	Average Depositional Lead			Average Airborne Lead Concentration		
	β ^1^	95% LCI, UCI		β	95% LCI, UCI	
**2008**						
Total (Direct + Indirect) Effect (TE)	0.21	0.20	0.23	0.20	0.18	0.21
Controlled Direct Effect (CDE)	0.16	0.14	0.18	0.14	0.12	0.15
Natural Direct Effect (NDE)	0.10	0.08	0.11	0.10	0.07	0.10
Natural Indirect Effect (NIE)	0.12	0.11	0.13	0.11	0.10	0.12
Percentage Mediated (PM)	54.42	50.34	58.50	56.55	52.28	60.82
Percentage Due to Interaction (PAI)	28.20	26.15	31.84	25.38	22.14	28.61
Percentage Eliminated (PE)	25.42	22.30	28.55	31.17	27.41	34.93
**2010**						
Total (Direct + Indirect) Effect (TE)	0.20	0.18	0.21	0.19	0.18	0.21
Controlled Direct Effect (CDE)	0.15	0.13	0.16	0.14	0.13	0.16
Natural Direct Effect (NDE)	0.12	0.10	0.12	0.11	0.10	0.12
Natural Indirect Effect (NIE)	0.09	0.08	0.10	0.09	0.08	0.09
Percentage Mediated (PM)	44.37	40.72	48.01	43.97	40.50	47.43
Percentage Due to Interaction (PAI)	18.90	16.13	21.67	17.58	14.57	20.60
Percentage Eliminated (PE)	25.47	22.30	28.63	26.38	23.01	29.76
**2011**						
Total (Direct + Indirect) Effect (TE)	0.12	0.10	0.13	0.12	0.11	0.13
Controlled Direct Effect (CDE)	0.12	0.11	0.13	0.11	0.10	0.13
Natural Direct Effect (NDE)	0.11	0.10	0.12	0.10	0.09	0.11
Natural Indirect Effect (NIE)	0.00 (ns)	−0.00	0.01	0.02	0.01	0.03
Percentage Mediated (PM)	2.77 (ns)	−2.74	8.29	16.37	11.98	20.76
Percentage Due to Interaction (PAI)	5.72 (ns)	1.19	10.25	8.97	4.43	13.50
Percentage Eliminated (PE)	−2.95 (ns)	−6.31	0.42	7.41	4.14	10.68

β ^1^ = causal effects and interactive effects of housing age, depositional lead and airborne lead concentrations on estimated childhood blood lead levels (BLLs) and 95% confidence intervals, controlling for child’s gender and method of laboratory specimen collection. (ns) = non-significant result. Average Depositional Lead (µg/m^2^) Average Airborne Lead Concentration (µg/m^3^) Lower Confidence Interval (LCI) Upper Confidence Interval (UCI).

**Table 5:** Causal Mediation and Interaction Effects of Older Housing (Built ≤ 1978) and Average Lead Deposition or Average Airborne Lead Concentrations on Childhood Blood Lead Levels, Controlling for Racial Residential Segregation, Detroit Metropolitan Area, 2008, 2010, 2011.

	Average Lead Deposition						Average Airborne Lead Concentration					
	β ^1^	95% LCI, UCI		β	95% LCI, UCI		β	95% LCI, UCI		β	95% LCI, UCI	
**2008**	**Black Children**			**White Children**			**Black Children**			**White Children**		
Total (Direct + Indirect) Effect (TE)	0.19	0.18	0.21	0.21	0.19	0.23	0.15	0.14	0.17	0.12	0.15	0.18
Controlled Direct Effect (CDE)	0.15	0.13	0.17	0.15	0.13	0.17	0.09	0.07	0.11	0.09	0.07	0.10
Natural Direct Effect (NDE)	0.08	0.06	0.09	0.09	0.08	0.11	0.03	0.01	0.04	0.04	0.02	0.05
Natural Indirect Effect (NIE)	0.12	0.11	0.13	0.12	0.11	0.13	0.13	0.12	0.14	0.13	0.12	0.14
Percentage Mediated (PM)	61.02	55.70	66.33	56.35	51.75	60.94	83.41	75.58	91.23	77.89	70.95	84.83
Percentage Due to Interaction (PAI)	25.54	22.59	28.49	31.24	27.97	34.51	30.81	26.77	34.86	35.39	31.10	39.68
Percentage Eliminated (PE)	23.47	19.54	27.40	29.32	25.76	32.88	42.25	35.84	48.66	46.07	40.14	51.99
**2010**	**Black Children**			**White Children**			**Black Children**			**White Children**		
Total (Direct + Indirect) Effect (TE)	0.18	0.17	0.19	0.17	0.16	0.19	0.176	0.16	0.19	0.18	0.17	0.12
Controlled Direct Effect (CDE)	0.13	0.11	0.14	0.13	0.11	0.14	0.124	0.11	0.14	0.12	0.11	0.14
Natural Direct Effect (NDE)	0.09	0.08	0.10	0.09	0.07	0.10	0.079	0.07	0.09	0.08	0.07	0.10
Natural Indirect Effect (NIE)	0.09	0.08	0.10	0.09	0.08	0.10	0.097	0.09	0.11	0.10	0.09	0.11
Percentage Mediated (PM)	49.38	44.94	53.81	50.61	45.99	55.23	55.28	50.58	59.97	53.70	49.26	58.15
Percentage Due to Interaction (PAI)	23.64	20.35	26.93	21.74	18.63	24.85	21.21	17.79	24.62	23.45	19.80	27.10
Percentage Eliminated (PE)	29.31	25.52	33.11	27.54	23.61	31.48	29.69	25.20	34.20	31.70	27.45	35.94
**2011**	**Black Children**			**White Children**			**Black Children**			**White Children**		
Total (Direct + Indirect) Effect (TE)	0.10	0.088	0.11	0.10	0.09	0.12	0.11	0.10	0.13	0.12	0.10	0.13
Controlled Direct Effect (CDE)	0.10	0.09	0.11	0.09	0.09	0.11	0.10	0.09	0.12	0.10	0.09	0.12
Natural Direct Effect (NDE)	0.09	0.08	0.10	0.09	0.08	0.11	0.08	0.07	0.09	0.08	0.07	0.09
Natural Indirect Effect (NIE)	0.01	0.01	0.02	0.01	0.01	0.02	0.04	0.03	0.04	0.04	0.03	0.04
Percentage Mediated (PM)	12.00	5.61	18.39	11.95	5.61	18.30	31.36	25.92	36.80	30.22	25.22	35.23
Percentage Due to Interaction (PAI)	9.44	4.23	14.65	9.79	4.41	15.18	13.69	9.16	18.23	16.82	11.47	22.18
Percentage Eliminated (PE)	1.97	−2.30	6.24	2.36	−1.87	6.57	11.95	7.09	16.81	15.14	10.95	19.33

β ^1^ = causal effects and interactive effects of housing age, lead deposition and airborne lead concentrations on estimated childhood blood lead levels (BLLs) and 95% confidence intervals, controlling for child’s gender and method of laboratory specimen collection Average Depositional Lead (µg/m^2^) Average Airborne Lead Concentration (µg/m^3^). Lower Confidence Interval (LCI) Upper Confidence Interval (UCI).

## Data Availability

Summary data used in this study are available upon request.

## Appendix A

The Michigan Air Emissions Reporting System (MAERS) requires that annual emission inventory reports be filed by commercial, industrial, or governmental entities for each permitted source (including grandfathered sources) contributing hazardous air pollutants and criteria pollutants (Michigan Rule 336) outlined by the Air Quality Division (AQD) policy and procedure number AQD-013 [53]. The monitoring and reporting emission threshold for lead in the state of Michigan is 0.5 tons per year (referenced in Michigan Rule 336.1212(7)). Allowable emissions are dictated by each facility’s specific individual permit [53]. Stack tests and Continuous Emission Monitoring Systems (CEMS) are used to collect emissions data [53]. The MAERS data (2006-2013) provided the variables for the lead emissions modeling described in the next section.

AERMOD is the U.S. EPA’s advanced plume modeling program with design criteria for regulatory applications [56]. It uses a gaussian approach to assume that the simulated concentration values follow a bell-shaped distribution where the concentration of the modeled pollutant is highest at the center of the point source’s plume and decreases exponentially approaching zero at the plume edge [62]. AERMOD incorporates air dispersion based on planetary boundary layer turbulence structure and scaling concepts, including treatment of both surface and elevated sources, and both simple and complex terrain. There are two input data preprocessors that are regulatory components of the AERMOD modeling system, AERMET and AERMAP. AERMET is a meteorological data preprocessor that incorporates air dispersion based on planetary boundary layer turbulence structure and scaling concepts using the AERMINUTE and AERSURFACE programs and data from the National Weather Service (NWS). AERMINUTE calculated hourly average wind speeds and directions.

Meteorological data and processing determine three surface characteristics: Surface roughness $\left\{z_{0}\right\}$, albedo {r}, and Bowen Ratio $\left\{b_{0}\right\}$. Surface roughness refers to the height of obstacles to the wind flow and its length is important in determining the magnitude of mechanical turbulence and stability of the boundary layer. The albedo is the fraction of total incident solar radiation reflected by the surface without absorption. The daytime Bowen Ratio is an indicator of surface moisture (ratio of sensible heat flux to latent heat flux). The albedo and Bowen Ratio are used to determine how much incoming radiation is converted to sensible heat flux. In this study, adjusted meteorological data corresponded to the year of emissions, i.e., 2006 meteorological data was applied to the 2006 lead emissions data. In 2015, the U.S. EPA proposed imputing meteorological data that has adjusted surface friction velocity and a low wind option to address issues with AERMOD model overprediction under stable, low wind speed conditions [62,63,64]. Particle deposition parameters were set at 0.75 for fine mass fraction and 0.5 for the representative mass-mean aerodynamic particle diameter in microns (U.S. EPA’s default values for lead). Surface characteristics parameters range from 0.16 to 0.47 for albedo, 0.49 to 0.96 for Bowen Ratio, and 0.018 to 0.090 for surface roughness length (km). The Detroit Metropolitan Airport (station id 94847) was utilized for surface weather variables and the national weather surface White Lake facility (station id 4830) for upper air variables.

The second preprocessor is AERMAP used to characterize terrain and generate receptor grids for AERMOD using the United States Geological Service (USGS) digital elevation data [62,63,64]. Surface characteristics are also determined using source of elevation, land use and land cover. In this study, urban versus rural modeling specification were selected. Surface roughness length is related to the height of obstacles to the wind flow and is, in principle, the height at which the mean horizontal wind speed is zero based on a logarithmic profile. When combined with wind speed, the effects are best modeled for stable atmospheric conditions and meteorological monitoring sites are typically open (low roughness) exposures to accommodate siting criteria [62,63,64]. The data used to determine the elevation (hills and terrain) and land cover were derived from the USGS’s 30-meter National Elevation Dataset (NED).

The domain of a 500 meter by 500-meter resolution was projected into UTM (Universal Transmercator Projection) zone 17 coordinate system. The plot file for the aggregate study period of 8 years (17,128 total hours) consisted of 21,136 grid receptors.

Predicted airborne and depositional lead concentrations are influenced by surface roughness parameter differences between the facility emission and the meteorological measurement site and will depend on the nature of the application (i.e., the release height, plume buoyancy, terrain influences, downwash considerations, design metric, etc.). Facility-identifying characteristics input into the modeling are provided in Moody and Grady [1].

In this study, emission quantities of greater than or equal to 0.1 pound (0.05 kg) per stack per year of atmospheric lead were modeled using AERMOD. Smaller quantities by certain facilities in certain years were initially input but found to be undetectable and therefore, not added in subsequent models. Because fugitive source emission heights were not provided at all, the authors used a standard industrial facility height of 10 meters (or 32.81 feet).

AERMOD’s first output for each receptor included the annual estimated airborne lead presented in μg/m^3^ and the total depositional lead reported in g/m^2^. AERMOD’s second output for each receptor included the monthly maximum levels for airborne lead obtained by dividing the annual pounds of lead emission by 8760 h (in a year) and then incorporating the hourly meteorology to derive maximum monthly and annual lead averages (direct hourly, monthly, and annual outputs are not automated in AERMOD for lead in particular).

In this study, both airborne (wet and dry) and depositional concentrations were mapped individually for each year of this study and compared temporally and across counties in the DMA. Some of the key assumptions applied to the dry deposition model included flux rate, concentration of lead, deposition velocity, height of stack, and surface roughness. The wet deposition model included the flux of the particulate, column average concentration of particles in the air, a washout coefficient, and precipitation rate from the meteorological profile [42]. Both the wet and dry flux were calculated on an hourly basis and summed to obtain total flux or deposition.

The average residence time of lead in the atmosphere is 10 days [13]; however, lead is extremely persistent in water and soil. The estimated lead emissions gridded-output values from AERMOD were input into ArcGIS v. 10.6 for each of the years of study [46]. The UTM coordinates were converted to a geographic coordinate system (latitude and longitude) to overlay onto addresses and census tracts or neighborhoods more easily.
